# Single-Nucleotide Polymorphisms as Biomarkers of Antipsychotic-Induced Akathisia: Systematic Review

**DOI:** 10.3390/genes14030616

**Published:** 2023-02-28

**Authors:** Regina F. Nasyrova, Elena E. Vaiman, Vera V. Repkina, Aiperi K. Khasanova, Azat R. Asadullin, German A. Shipulin, Kuanysh S. Altynbekov, Mustafa Al-Zamil, Marina M. Petrova, Natalia A. Shnayder

**Affiliations:** 1 Institute of Personalized Psychiatry and Neurology, V.M. Bekhterev National Medical Research Center for Psychiatry and Neurology, 192019 Saint Petersburg, Russia; 2 Department of Psychiatry, Russian Medical Academy for Continual Professional Education, 125993 Moscow, Russia; 3 Department of Psychiatry and Addiction, Bashkir State Medical University, 450008 Ufa, Russia; 4 Postgenome Technologies Center, Center for Strategic Planning of FMBA of Russia, 119121 Moscow, Russia; 5 Republican Scientific and Practical Center of Mental Health, Almaty 050022, Kazakhstan; 6 Department of Psychiatry and Narcology, S.D. Asfendiarov Kazakh National Medical University, Almaty 050022, Kazakhstan; 7 Department of Physiotherapy, Peoples’ Friendship University of Russia, 117198 Moscow, Russia; 8 Shared Core Facilities “Molecular and Cell Technologies”, V.F. Voino-Yasenetsky Krasnoyarsk State, Medical University, 660022 Krasnoyarsk, Russia

**Keywords:** antipsychotic-induced akathisia, adverse drug reaction, extrapyramidal disorder, antipsychotics, pharmacogenetics, gene, genetic biomarker, single nucleotide variant, association, variation

## Abstract

Antipsychotic-induced akathisia (AIA) is a movement disorder characterized by a subjective feeling of inner restlessness or nervousness with an irresistible urge to move, resulting in repetitive movements of the limbs and torso, while taking antipsychotics (APs). In recent years, there have been some associative genetic studies of the predisposition to the development of AIA. **Objective**: The goal of our study was to review the results of associative genetic and genome-wide studies and to systematize and update the knowledge on the genetic predictors of AIA in patients with schizophrenia (Sch). **Methods:** We searched full-text publications in PubMed, Web of Science, Springer, Google Scholar, and e-Library databases from 1977 to 2022. The Preferred Reporting Items for Systematic Reviews and Meta-Analyses (PRISMA) quality scale was used for the critical selection of the studies. **Results:** We identified 37 articles, of which 3 were included in the review. Thus, the C allele of rs1800498 (59414 C>T) and the A allele of rs1800497 (17316 G>A) (TaqIA) from the *DRD2* gene as well as the TT genotype rs13212041 (77461407 C>T) from the *HTR1B* gene were found to be associated with AIA. **Conclusions**: Uncovering the genetic biomarkers of AIA may provide a key to developing a strategy for the personalized prevention and treatment of this adverse neurological drug reaction of APs in patients with Sch in real clinical practice.

## 1. Introduction

Akathisia is a neurological movement disorder characterized by a subjective feeling of inner restlessness or nervousness with an irresistible urge to move, resulting in repetitive movements such as crossing legs, swaying, or constantly switching from one leg to another [1,2]. The first description in the literature dates back to 1901 when the Czech neuropsychiatrist Ladislav Gaskovec described a phenomenon that he called “inability to sit”, which was a non-drug related akathisia [3]. The first report of drug-induced akathisia appeared only in 1960 when Kruse W. described three patients who developed “muscle restlessness” while taking phenothiazines (a group of antipsychotics (APs)) [4]. Akathisia as a symptom can be a part of both hereditary and acquired neurodegenerative diseases (Appendix A, Table A1).

The most common form of secondary akathisia is drug-induced akathisia [5]. Drug-induced akathisia can develop while taking drugs of various pharmacological groups (Appendix A, Table A2). However, this drug-induced neurological complication most often develops while taking APs, including first-generation APs (haloperidol and chlorpromazine) and second-generation APs (risperidone, olanzapine, sulpiride, ziprasidone, quetiapine, clozapine, aripiprazole, and amisulpiride) [5].

**Figure A1:**
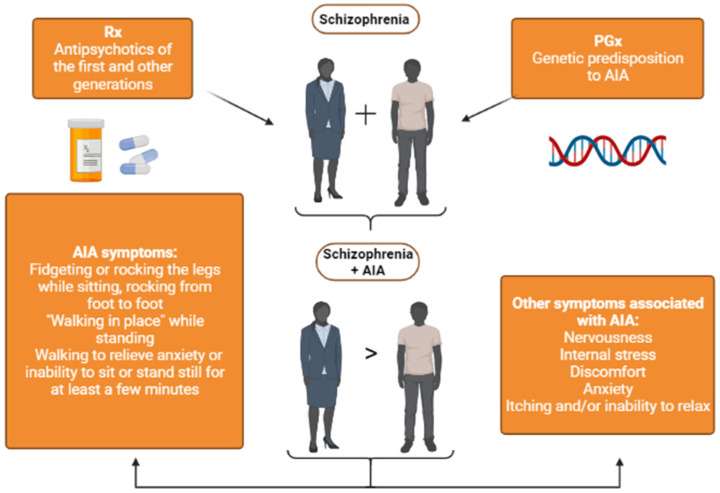
The clinical picture of antipsychotic-induced akathisia (AIA) in patients with schizophrenia (Sch). Note: Rx-prescription and PGx-pharmacogenetics.

Therefore, antipsychotic-induced akathisia (AIA) is a movement disorder occurring while taking APs and is characterized by a subjective feeling of inner nervousness with an irresistible urge to move (Appendix A, Figure A1) [1,2].

The prevalence of AIA among adult patients with schizophrenia (Sch) varies widely between 0.85% and 55% around the world, while the average prevalence of AIA worldwide is nearly 30% [6]. In addition to taking APs, there are non-modifiable and modifiable risk factors for the development of AIA, as presented in Appendix A, Table A3.

Thus, AIA is a multifactorial neurological disorder in which both a genetic predisposition and environmental factors play a role. Non-modifiable risk factors for the development of AIA include female sex (for tardive AIA); middle age; and genetic predisposition. In addition, modifiable factors pose a high risk in the development of AIA, such as long-term use of APs; vitamin B6 deficiency; ferritin deficiency; low serum iron; traumatic brain injury; alcohol abuse; autoimmune NMDAR-encephalitis; and cancer. The genetic predisposition to the development of AIA involves the carriage of single nucleotide polymorphisms (SNPs) of genes, which are under the influence of external modifying factors. Based on statistical data, the incidence of SNPs in the population is 3%, thus, when also subject to risk factor conditions, the possibility of AIA increases significantly. The role of SNPs in the mechanism of AIA development has been evaluated for more than 40 years and the number of publications revealing the significance of SNPs in the pathogenesis of AIA continues to increase [7,8,9,10,11]. Thus, the study of the SNPs of other candidate genes associated with AIA in patients with Sch is relevant [12,13,14,15,16].

The aim of this study was to identify the relevant SNPs/polymorphisms of candidate AIA genes since, based on these data, it is possible to compile a genetic risk panel for the chances of patients with Sch developing AIA.

## 2. Materials and Methods

### 2.1. Search Strategy

We searched full-text publications in PubMed, Web of Science, Springer, Google Scholar, and e-Library databases in English and Russian. The keywords were as follows: antipsychotic-induced akathisia; drug-induced akathisia; antipsychotics; genes; adverse drug reaction; extrapyramidal disorder; antipsychotics; pharmacogenetics; genetic biomarker; single nucleotide variant; association; variation; and akathisia genes. In addition, earlier publications of historical interest were included in the review.

### 2.2. Eligibility Criteria

We analyzed placebo-controlled studies, cross-sectional studies, case-control studies, case studies, systematic reviews, meta-analyses, and Cochrane reviews from 1977 to 2022. Duplicate articles were excluded from the analysis.

### 2.3. Review Strategy 

The analyzed data were presented as text and divided by genes. Further, the data are summarized in Table 1, considering genes, SNPs, and the presence or absence of an association with the risk of developing AIA.

### 2.4. Data Synthesis 

**Figure 1:**
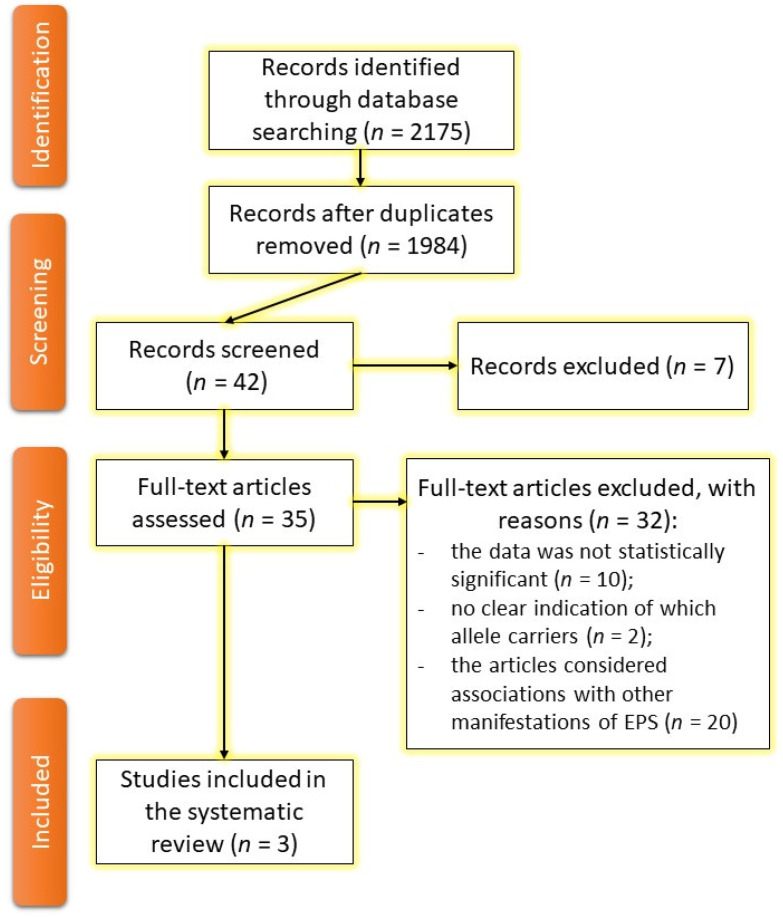
PRISMA chart flow.

The search was conducted throughout the year from 10 October 2021 to 19 September 2022 by double independent peer review. A total of 2175 articles were found by keywords. After exclusion, we analyzed 35 articles, and only 3 articles were found to match the search. We excluded articles in which the data were not statistically significant; the SNPs data did not match the international nomenclature databases; associations with specific alleles/polymorphisms were not indicated in the articles; samples included less than 100 people; and the articles considered associations with other manifestations of AP-induced extrapyramidal syndrome (EPS). The search data are presented in a Preferred Reporting Items for Systematic Reviews and Meta-Analyses (PRISMA) chart flow (Figure 1) [17]. The register number is CRD42022374137.

### 2.5. Data Not Included in the Review

We excluded 20 studies that showed positive associations with AP-induced movement disorders without differentiating with which ones [13,18,19,20,21,22,23,24,25,26,27,28,29,30,31,32,33,34,35,36].

We also excluded the studies where there was no clear indication of which allele carriers and which SNPs had a high risk of developing AIA, as well as studies where the presented data did not match the international nomenclature databases [37,38].

Lastly, we excluded studies with statistically insignificant data [39,40,41,42,43,44,45,46,47,48].

## 3. Results

### 3.1. Genes Encoding Key Enzymes in Metabolism of Antipsychotics

**Figure A2:**
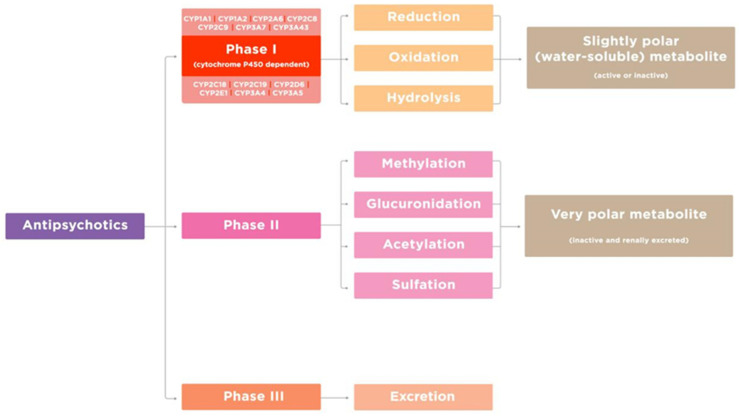
Antipsychotic metabolism phases [49].

Cytochrome P450 (CYP) enzymes of the liver are involved in the metabolism of more than 85% APs [49]. Many APs undergo several sequential biotransformation reactions. Biotransformation is catalyzed by specific enzyme systems which may also catalyze the metabolism of endogenous substances such as steroid hormones. The liver is the major site of biotransformation, although specific APs may undergo biotransformation primarily or extensively in other tissues [50]. Most often, APs biotransformation reactions occur in the liver, however, individual APs undergo these reactions to a greater or lesser extent in other organs and tissues of the human body [49]. APs metabolized via phase I reactions have longer half-lives (Appendix A, Figure A2) [49].

**Figure A3:**
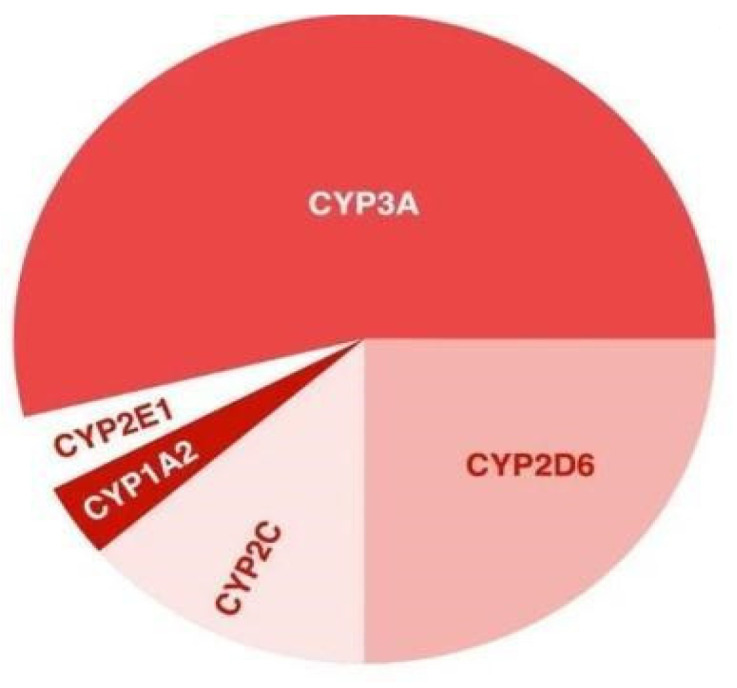
Estimated percentage of antipsychotics metabolized by the major cytochrome P450 enzymes [49].

Enzymes catalyzing this phase biotransformation are mostly from the cytochrome P450 system, flavin-containing monooxygenase system, monoamine oxidase, aldehyde and alcohol dehydrogenase, deaminases, esterases, amidases, and epoxide hydratases [51,52]. Oxidation reactions, which occur with CYP enzymes (mixed function oxidases (MFO) or mono-oxygenases), take place in the smooth endoplasmic reticulum (ER) of the cell [53]. These reactions involve cytochrome P450 reductase, nicotinamide adenine dinucleotide phosphate (NADPH), and oxygen (O2). CYP enzymes also better metabolize APs with a high-fat solubility [52]. The CYP system is involved in numerous reactions, for example, hydroxylation; dealkylation; deamination; sulfoxidation; and oxidation [54]. The isoenzymes of the main APs metabolism pathways currently studied in the treatment of Sch are CYP1A2, CYP2C9, CYP2C19, CYP2D6, and CYP3A4 (Appendix A, Figure A3) [49]. 

Most often, CYP enzymes are in the liver, but they are also founded in other organs and tissues of the human body (for example, in the small and large intestines, testicles or ovaries, duodenum, pancreas, kidneys, spleen, lymph nodes, etc.). The enzymes of the CYP system are in the endoplasmic reticulum in cells. The four phenotypes are distinguished, depending on the metabolic activity of isoenzymes, as extensive (EM—extensive metabolizers), intermediate (IM—intermediate metabolizer), poor (PM—poor metabolizer), and ultrafast (URM—ultrarapid metabolizers) and they are characterized by a normal, intermediate, reduced, and increased ability to metabolize enzyme substrates, respectively [55]. Carrying low-functional or non-functional SNPs of the genes encoding the hepatic cytochrome of P450 isoenzymes in patients with PM phenotype can greatly affect the metabolic rate of AP in one or more metabolic pathways, or AP with narrow dose ranges, such as haloperidol [56,57,58] (Appendix A, Table A4 and Table A5).

### 3.2. Genes Encoding the Transport Proteins of Antipsychotics (via the Blood-Brain Barrier)

The transcellular transport of biologically active substances via the blood-brain barrier (BBB) can be carried out in the following ways [59,60]: simple diffusion; facilitated diffusion; endocytosis via receptor-mediated transcytosis; and efflux transport. Efflux is the active removal of a substance from a cell through a protein pump embedded in the cell membrane. Efflux transport is movement in the “brain-blood” direction [59]. 

In recent years, much more attention has been paid to studies of this transcellular transport pathway across the BBB [61,62]. The most important transport efflux mechanism is believed to be the carrier-mediated excretion of APs from the brain to blood. BBB endothelial cells contain numerous membrane transporters involved in the influx or efflux of various major substrates such as electrolytes, nucleosides, amino acids, and glucose [15,61,63]. Efflux transport is based on the so-called ATP-Binding Cassette (ABC) transport proteins associated with ATP [15,63]. ABC transport proteins have an affinity for a broad category of solutes, especially for large fat-soluble molecules with a number of nitrogen and oxygen atoms in their structure. These ABC transport proteins use ATP hydrolysis to pump molecules across the membrane and, hence, they can cause solute efflux against a concentration gradient [15,64,65]. P-glycoprotein (P-gp: ABCB1) and breast cancer-associated protein (BCRP: ABCG2) are the main transporters of ABC efflux in the BBB [62,66,67,68,69].

Active transport proteins of APs efflux across the BBB from the ABC family are increasingly recognized as important determinants of APs’ distribution in the central nervous system (CNS) and their excretion [59,66]. The P-gp, as a transport protein, has shown itself to be a key element of the BBB in most people. It can actively transport a huge number of lipophilic drugs from the endothelial cells of the brain capillaries that form the BBB. In addition to P-gp, other transporter proteins, such as members of the multidrug resistance protein (MRP) family and BCRP, appear to contribute to APs’ efflux across the BBB [70]. 

The implications of all these transport proteins at the BBB level include the minimization or prevention of AP-induced neurotoxic adverse drug reactions (ADRs) [15,64,65], aggravation of Sch symptoms [56,64], or development of pseudo resistance to APs [65,71]. At the same time, ABC transport proteins may also limit the central distribution of the APs used to treat Sch, increasing the risk of developing therapeutic resistance [64,65,67].

Therefore, knowledge of the genetically determined changes in the functional activity and expression of the aforementioned BBB transport proteins can help form a new personalized strategy for predicting the elimination of APs from the brain and provide new therapeutic opportunities for therapeutically resistant Sch (Appendix A, Table A6).

The most studied and clinically significant transport proteins provide APs’ efflux across the BBB and the membrane of target neurons of APs’ action [68,72] (Appendix A, Table A7). 

In the case of a genetically determined decrease in the functional activity or expression of the P-gp, BCRP, and Multidrug Resistance-Associated Protein 1 (MRP1) transport proteins at the level of the BBB endothelial cell membranes, the APs’ efflux from the brain into the blood is disturbed to varying degrees (decreases significantly, insignificantly, or moderately) [68]. This, in turn, leads to an increase in the exposure time of these APs to the brain and an increased risk of cumulation during chronic (long-term) psycho pharmacotherapy, and significantly raises the risk of developing serious AP-induced neurotoxic ADRs [64]. The accumulation of APs ultimately leads to a slowdown in their metabolism due to the enzymatic system, and therefore the phenotype of such patients with Sch is more often referred to as a PM rather than a poor transporter [73].

Appendix A, Table A6 shows that the SNPs of the *ABCB1* gene are of the greatest clinical interest in psychiatric practice since the P-gp encoded by this gene is involved in the efflux through the BBB of many APs. The least studied is the *ABCC1* gene, whose role in pharmacogenetics (PGx) continues to be actively studied. On the other hand, some APs (e.g., clozapine, olanzapine, quetiapine, etc.) are cleared through the BBB by several transporter proteins, which is considered important when clinically interpreting the results of PGx in real psychiatric practice. This demonstrates that a comprehensive approach to assessing the contribution of the SNPs of these genes that affect the APs efflux of the first and new generations is relevant and scientifically substantiated.

### 3.3. Genes Encoding Targets of Antipsychotics

#### 3.3.1. Key Receptors for Antipsychotics Action

Many pharmacogenetic studies have confirmed the clinical validity and importance of some brain neurotransmitter systems in mediating treatment efficacy and the onset of ADRs. The genetic variability of dopaminergic and serotoninergic receptors plays a significant role in APs’ efficacy [55]. Based on the fact that a dopaminergic receptor blockade is the leading theory for the development of AIA, the genes of the dopaminergic system are key targets [74]. At the same time, the pathogenesis of AIA is complex and the serotonergic and glutamatergic systems should also be considered. Thus, the genes of the serotonergic and glutamatergic systems are also key targets [75] (Appendix A, Table A8).

#### 3.3.2. Key Enzymes of Antipsychotics Action 

Key enzymes are represented by genes encoding Heparan Sulfate Proteoglycan 2 (*HSPG2*), catechol-O-methyltransferase (*COMT*), NAD(P)H Quinone Dehydrogenase 1 (*NQO1*), the Regulator of G Protein Signaling 2 (*RGS2*), Glutathione S-Transferase Pi 1 (*GSTP1*), Protein Phosphatase 1 Regulatory Inhibitor Subunit 1B 9 *PPP1R1B*), Brain-Derived Neurotrophic Factor (*BDNF*), and Manganese-containing superoxide dismutase (*MnSOD*) (Appendix A, Table A9).

### 3.4. Evidence from a Systematic Review

Based on the results of a systematic review, we selected three studies that met the Materials and Methods selection criteria; these studies are presented in Table 1.

**Table 1:** Systematic review results.

Gene (OMIM * Number)	SNP (Location)	Association with AIA	*p*-Value	Sample	Country	Reference
*DRD2* (126450)	rs1800498 (NG_008841.1:g.59414C>T) (TaqI_D)	Major allele C is associated with the risk of AIA	0.001	402	The Netherlands	[76]
	rs1800497 (NG_012976.1:g.17316G>A) (TaqIA)	Minor allele A is associated with the risk of AIA	0.03	402	The Netherlands	[76]
			0.011	234	Australia	[77]
*HTR1B* (182131)	rs13212041 (NC_000006.12:g.77461407C>T)	Homozygous genotype TT is associated with the risk of AIA	0.004	229	Croatia	[75]

Note: * from the open database OMIM—Online Mendelian Inheritance in Man [78]; SNP—single-nucleotide polymorphism; and AIA—antipsychotic-induced akathisia.

#### 3.4.1. The *DRD2* Gene

**Figure A4:**
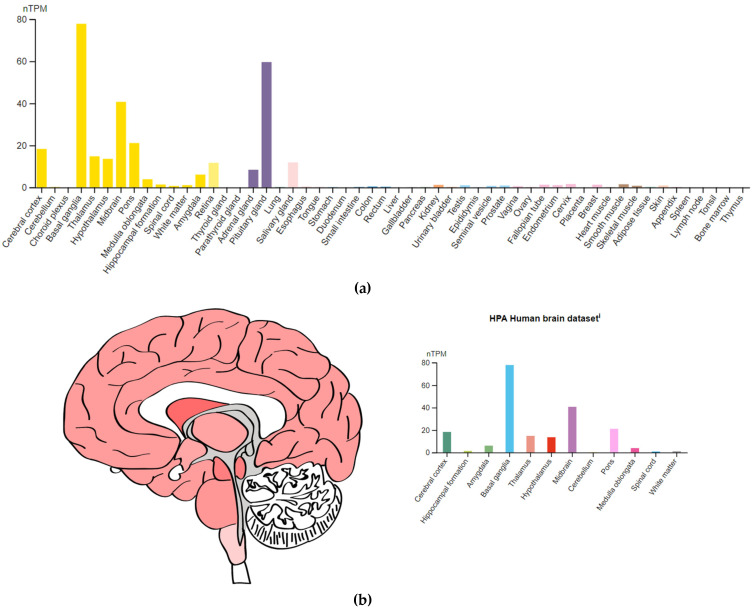
Expression of the *DRD2* gene in the human body (**a**) and the brain (**b**) [81].

The *DRD2* gene is located on the 11q23.2 chromosome and encodes D2-type dopaminergic receptors [79,80]. This gene is predominantly expressed in the brain, most commonly in the basal ganglia, midbrain, cerebral cortex, and pons (Appendix A, Figure A4) [7,81,82].

The D2 receptors are members of the G protein-coupled dopamine receptor family, which also includes D1, D3, D4, and D5 receptor types [83]. They are involved in the modulation of locomotion, reward, reinforcement, memory, and learning. The D2 receptor inhibits the activity of the adenylate cyclase. Abnormalities in the structure of the *DRD2* gene have been associated with affective disorders [84] and with peak dose dyskinesia in patients with Parkinson’s disease (PD) [85]. A missense mutation in this gene can presumably cause myoclonic dystonia. Other SNPs have been described in patients with Sch. Alternative splicing of the *DRD2* results in two transcript variants encoding different isoforms. A third variant has been described, but it has not been determined whether this form is normal due to aberrant splicing or not [81,86].

We found two studies of SNPs of the *DRD2* gene with a risk of developing AIA. Koning et al. [76] studied the association of thirteen SNPs of nine candidate genes (*DRD2, DRD3, 5HTR2A, 5HTR2C, COMT, NQO1, GSTP1, RGS2,* and *MnSOD*) with the risk of developing AIA in 402 Northern European patients with mental disorders taking APs for at least a month. Positive statistically significant associations were found with allele C of rs1800498 (NG_008841.1:g.59414C>T) (TaqI_D) (*p*-value *=* 0.001) and allele A of rs1800497 (NG_012976.1:g.17316G>A) (*p*-value = 0.03). Other authors also confirmed that minor allele A of rs1800497 (NG_012976.1:g.17316G>A) (TaqIA) (*p*-value = 0.011) is associated with the risk of developing AIA according to the Barnes Akathisia Rating Scale (BARS) among 234 Australian patients with mental disorders on AP monotherapy for at least a month (*p*-value = 0.011) [77]. 

#### 3.4.2. The *HTR1B* Gene

**Figure A5:**
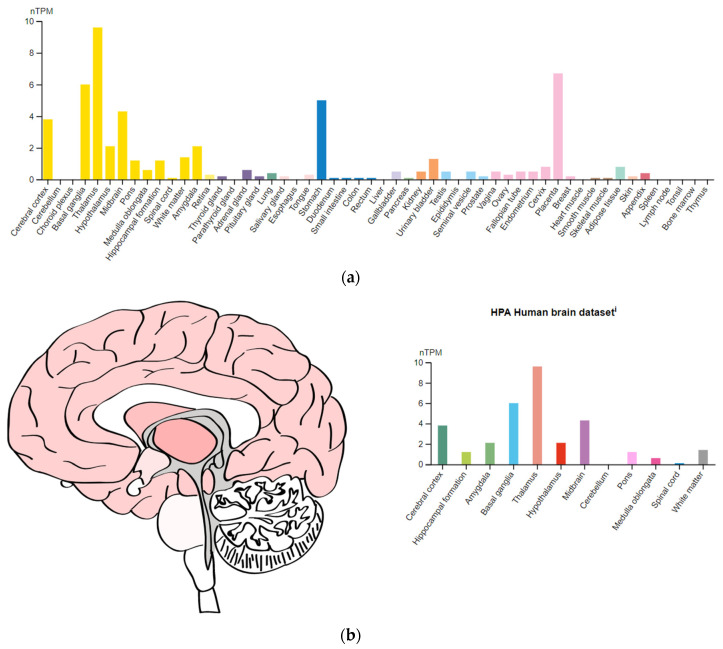
Expression of the *HTR1B* gene in the human body (**a**) and the brain (**b**) [122].

The *HTR1B* gene is located on the 6q14.1 chromosome and encodes a G-protein-coupled 5-hydroxytryptamine (serotonin) receptor. The gene is expressed in the brain, mainly in the thalamus and basal ganglia (Appendix A, Figure A5) [87].

The protein functions as a receptor for ergot alkaloid derivatives, various anxiolytics and antidepressants, and other psychoactive substances such as lysergic acid diethylamide. It also regulates the release of 5-hydroxytryptamine, dopamine, and acetylcholine in the brain and thereby influences neural activity, nociceptive processing, pain perception, mood, and behavior. In addition, it plays a role in the vasoconstriction of cerebral arteries [88].

In the study, the authors studied the association between five SNPs (rs6313, rs3813929, rs6295, rs13212041, and rs1805054) of the candidate genes (*HTR1A*, *HTR1B*, *HTR2A*, *HTR2C*, and *HTR6*) with the risk of developing AIA. As a result, a positive association was noted between the carriage of the homozygous genotype TT of rs13212041 (NC_000006.12:g.77461407C>T) of the *HTR1B* gene with the risk of AIA in patients with Sch according to the BARS scale (*p*-value = 0.004) [75].

## 4. Discussion

### 4.1. Summary of Evidence

**Figure 2:**
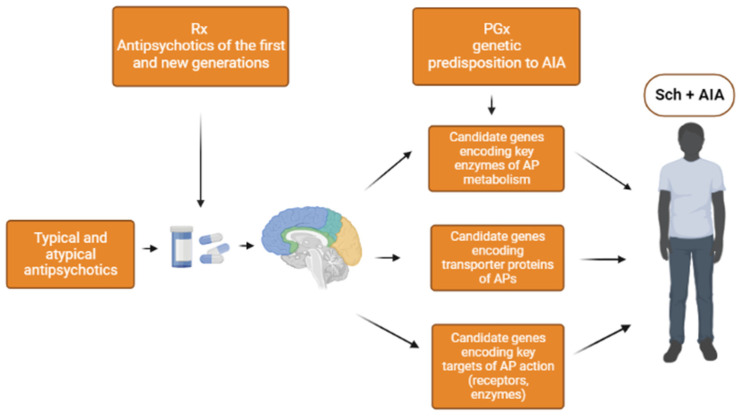
Summary scheme of the genetic predisposition to antipsychotic-induced akathisia (AIA) in patients with schizophrenia (Shc). Note: Rx-prescription and PGx-pharmacogenetics.

Our review of the potential genetic biomarkers of AIA made it possible to systematize the results of previous associative genetic studies. The greatest interests for researchers concerning the genetic biomarkers of AIA, including the candidate genes: *GRIN2A, GRIN2B, HSPG2, DRD2, DRD3, DRD4, COMT, HTR2A, HTR2C, PPP1R1B, BDNF, MnSOD (SOD2),* and *GSTP1* (Figure 2).

In this review, with the help of Table 1, we visually displayed the available data in terms of genetic biomarkers for the development of AIA in patients with Sch, despite the small number of articles included.

Although we conducted a comprehensive search of frequently used databases and search terms, it cannot be ruled out that some recent publications may have been overlooked. According to the results of our systematic review, only three studies could be included that confirmed the carriage of SNPs in the studied candidate genes: C allele of rs1800498 (NG_008841.1:g.59414C>T) (TaqI_D) and A allele of rs1800497 (NG_012976.1:g.17316G>A) (TaqIA) of the *DRD2* gene and the TT genotype of rs13212041 (NC_000006.12:g.77461407C>T) of the *HTR1B* gene. 

### 4.2. Comparison with the Existing Literature 

Basically, the SNPs which we reviewed were studied in the framework of associative studies with mental disorders. At the same time, it was discovered that the SNPs of some of the discussed candidate genes are associated not only with the development of AIA but also with antipsychotic-induced parkinsonism (AIP). Thus, rs1800497 (Taq1A) of the *DRD2* gene is associated with a high risk of AIP [22,89]. 

Among the genes studied for encoding the P450 isoenzymes, involved in the metabolism of APs through the hepatic metabolic pathway, related to the development of AIA were the *CYP1A2* gene (responsible for the metabolism of haloperidol, thioridazine, thiothixene, trifluoperazine, flupentixol, chlorpromazine, loxapapine, promazine, asenapine, zotepine, quetiapine, clozapine, lumateperone, olanzapine, perphenazine, and pimozide [76]), and the *CYP2D6* gene (responsible for the metabolism of alimemazine, promazine, zuclopenthixol, thioridazine, haloperidol, trifluoperazine, levomepromazine, flupentixol, perphenazine, fluphenazine, pipothiazine, chlorpromazine, and prochlorperazine [90]).

It is known that other cytochrome P450 isoenzymes are also involved in the metabolism of APs, including *CYP1A1* (haloperidol, olanzapine, and perispirone); *CYP1B1* (perphenazine); *CYP2A6* (promazine and clozapine); *CYP2B6* (quetiapine); *CYP2C8* (perphenazine, clozapine, lumateperone, and perospirone), *CYP2C9* (haloperidol, perphenazine, promazine, clozapine, and olanzapine); *CYP2C18* (perphenazine); and *CYP2C19* (haloperidol, pipotiazine, perphenazine, promazine, and thioridazine) [90]. In addition, several polymorphisms of the *CYP2D6* gene are associated with a high risk of AIA [91,92,93]. 

However, we did not find any studies highlighting the role of non-functional and/or low-functional alleles of SNPs of the genes encoding the liver P450 isoenzymes on the development of AIA in patients with Sch. It is noteworthy that the greatest interest in the study of genetic biomarkers of AIA is among researchers in the Northern European countries, where this problem is being actively studied. In other regions of the world, including Russia, genetic studies of AIA have not been found. However, based on the data, we could not define whether the authors studied the family history of the observed patients with AIA for monogenic neurodegenerative diseases in which akathisia can be part of the syndrome of the disease, such as Huntington’s disease, hereditary kinesiogenic and non-kinesiogenic dyskinesias, etc. [94].

The risk of AIP increases depending on the minor allele of some SNPs and haplotypes of the candidate genes regulating G-protein signaling, including the *RGS2* gene (regulator of G-protein signaling 2); the *ADORA1* gene (adenosine A1 receptor); and the *ADORA2A* (adenosine A2A receptor) and *ADORA3* (adenosine A3 receptor) genes. However, the authors did not find any documented associations of the SNPs of these genes with changes in the risk of developing AIA [33,95,96].

Clinical manifestations of AIA lead to a significant decrease in the quality of life of patients with Sch and a decrease in their compliance with psychopharmacotherapy [78]. This explains the need for further research aimed at searching for the genetic biomarkers of AIA in various racial and ethnic groups of psychiatric patients. The planning of large single-center and multicenter studies adopting a standardized design seems to be required. Furthermore, future studies should concentrate on including ethnically and racially heterogeneous populations of Russia and other countries.

## 5. Limitation

Regarding limitations, we would like to highlight the low quality of the studies we reviewed. We considered interesting associative data, however; when using the databases of The National Center for Biotechnology Information (NCBI) [97,98] as controls, the data were inconsistent, in particular, other alleles were presented in particular SNPs that were not even complimentary. 

At the same time, some authors considered the association of EPS with specific SNPs as a single disease without differentiation into the main AP-induced extrapyramidal disorders, in particular AIA, AIP, and AP-induced tardive dyskinesia.

## 6. Conclusions

It should be recognized that there is no final or single decision on the leading role of any SNPs/polymorphisms of candidate genes in the development of AIA. Uncovering the genetic predictors of AIA (as the most common neurological ADRs in the treatment of patients with Sch) may provide a key to developing a strategy for the personalized prevention and treatment of AP-induced extrapyramidal complications in patients with Sch in real clinical practice. However, to confirm this theory, there is a need for larger multicenter studies with different racial and ethnic groups of patients. Of course, currently, there are publications of studies of candidate genes leading to the development of AIA, but at the same time, clear genetic biomarkers are not known. There is no unifying research on this topic of interest.

## Data Availability

Not applicable.

## Appendix A

**Table A1:** Primary and secondary akathisia.

Primary Akathisia	Secondary Akathisia
Huntington’s disease	Drug-induced akathisia
Hereditary kinesiogenic dystonia	Sindengham`s disease
Hereditary non-kinesiogenic dystonia	Symptomatic Tourette’s syndrome
Familial Tourette syndrome	

**Table A2:** List of drugs which predispose patients to drug-induced akathisia.

Drug	Frequency of Akathisia	Mechanism	Level of Evidence	References
A. Antipsychotics				
1. First-generation antipsychotics				
Haloperidol	24.8%	Blockade of dopaminergic D2 receptors in the limbic system and basal ganglia	A	[99]
Chlorpromazine	15.9%		A	
2. Second-generation antipsychotics				
Amisulpride	11.3%	Blockade of serotonergic 5-HT2, dopaminergic D2, and adrenergic receptors in the limbic system and basal ganglia	A	[100]
Aripiprazole	8.5%		A	[101]
Ziprasidone	8.3%		A	
Clozapine	7.9%		A	[102]
Quetiapine	5.2%		A	[101]
Olanzapine	8.7%		A	
Paliperidone	3.3%		A	
Risperidone	14.2%		A	[103]
Sulpiride	16.4%		A	[101]
B. Antidepressants				
Selective serotonin reuptake inhibitors				
Fluoxetine	NA	Blockade of serotonergic 5-HT2C receptors	C	[104]
Sertraline	NA	Dopamine inhibition through its high-affinity sodium-dependent reuptake in presynaptic endings	C	
Paroxetine	NA	Blockade of dopaminergic D2 receptors	C	
Fluvoxamine	NA	Blockade of serotonin reuptake, increased action of serotonin on 5HT1A autoreceptors	C	
Escitalopram	NA	Blockade of dopaminergic D2 receptors	C	
2. Selective serotonin and norepinephrine reuptake inhibitors				
Venlafaxine	NA	Dopamine inhibition through its high-affinity sodium-dependent reuptake in presynaptic endings	C	[104]
Duloxetine	NA		C	
3. Tricyclic antidepressants				
Mirtazapine	NA	Blockade of serotonergic 5-HT2- and 5-HT3-receptors as well as α2-adrenergic receptors	C	[105]
C. Antibacterial drug				
Azithromycin	NA	Unknown	C	[106]
Clarithromycin	NA		C	[107]
D. Calcium channel blockers				
Amlodipine	NA	Receptor hypersensitivity in the substantia nigra	C	[108]
Flunarizine	NA	Unknown	C	[109]
E. Benzodiazepines				
Clonazepam	NA	Agonist of GABA receptors	C	[110]
Clorazepate	NA		C	
Lorazepam	NA		C	
F. β1-adrenergic antagonists				
Betaxolol	NA	Unknown	A	[111]
G. β2-adrenergic antagonists				
Propanolol	NA	Blockade of serotonergic 5-HT1A and 5-HT1B receptors	A	[112]
H. Anticonvulsants				
Pregabalin	NA	Unknown	C	[113]
Lamotrigine	NA	Blockade of dopaminergic D2 receptors	C	[114]
I. Excitants				
MDMA (Ecstasy)	NA	Blockade of dopaminergic and serotonergic 5-HT receptors, a decrease in the number of 5-HT receptors	C	[115]
Cocaine	NA	Dopamine reuptake inhibition	C	[116]
J. Antimycotics				
Ciprofloxacin	NA	Unknown	C	[111]
K. Dopaminomimetics				
L-DOPA	NA	Increased activity of the direct dopaminergic striatal pathway	C	[117]
L. Lithium preparations				
Lithium	NA	Activation of dopaminergic neurotransmission	C	[118]
M. Centrally acting dopamine receptor antagonist				
Metoclopramide	20–25%	Blockade of dopaminergic D2 receptors	B	[119]

Notes: GABA-γ-aminobutyric acid, MDMA-methylenedioxymethamphetamine, NA-not available; A-Level I evidence or sustained multiple Level II, III, or IV evidence, data from one or more randomized, controlled clinical trials; B-Level II, III, or IV, evidence considered generally strong, data from non-randomized clinical trials, prospective observational studies, cohort studies, retrospective studies, case-control studies, meta-analyses and/or post-marketing observations; C-Level II, III, or IV, evidence, but the evidence is generally unstable, publication data of one or more clinical cases or series of cases [120].

**Table A3:** Risk factors for antipsychotic-induced akathisia.

Non-Modifiable Risk Factor	Modifiable Risk Factor
Second period of middle age (male: 36–60 years; female: 36–55 years) *	Reception APs: - duration of admission; - high daily dose
Female (for tardive AIA)	Brain injury
Caucasians	Malignant neoplasms
Genetic predisposition	Ferritin deficiency Low serum iron
	Alcohol abuse
	Vitamin B6 deficiency
	Autoimmune NMDAR encephalitis

Note: AIA-antipsychotic-induced akathisia, APs-antipsychotics, NMDAR-ionotropic glutamate receptor, and * age periods according to WHO classification [121].

**Table A4:** Candidate genes encoding key enzymes in antipsychotic metabolism.

Gene (OMIM Number *)	Chromosome Location **	Genomic Coordinate **	Protein ***
*CYP1A2* (124060)	15q24.1	chr15:74,748,845–74,756,607(GRCh38/hg38)	Cytochrome P450 Family 1 Subfamily A Member 2
*CYP2C9* (601130)	10q23.33	chr10:94,938,658–94,990,091(GRCh38/hg38)	Cytochrome P450 Family 2 Subfamily C Member 9
*CYP2C19* (124020)	10q23.33	chr10:94,762,681–94,855,547(GRCh38/hg38)	Cytochrome P450 Family 2 Subfamily C Member 19
*CYP2D6* (124030)	22q13.2	chr22:42,126,499–42,130,865(GRCh38/hg38)	Cytochrome P450 Family 2 Subfamily D Member 6
*CYP3A4* (124010)	7q22.1	chr7:99,756,960–99,784,248(GRCh38/hg38)	Cytochrome P450 Family 3 Subfamily A Member 4

Note: * from the open database OMIM—Online Mendelian Inheritance in Man [78], ** from the open database GeneCards: The Human Gene Database [86], *** from the open database The Human Protein Atlas [81].

**Table A5:** Antipsychotics with hepatic and predominantly hepatic metabolism.

Antipsychotic	Metabolism in the Liver	Enzyme of Cytochrome P450
A. First-Generation Antipsychotics		
Chlorpromazine	Hydroxylation N-dealkylation	CYP2D6 (the main path), CYP1A2, and CYP3A4
Haloperidol	Glucuronization N-dealkylation	CYP2D6, CYP3A, and CYP1A2
Perphenazine	Oxidation, N-dealkylation	CYP2D6, CYP1A2, and CYP3A4
Thioridazine	Betaoxidation N-dealkylation	CYP2D6 and CYP1A2
Flupentixol	Sulfonic acidification N-dealkylation Glucuronization	CYP2D6
Tiapride	Oxidation (up to 15%)	Underexplored
Sulpiride	Not metabolized, excreted through the kidneys (about 95%)	Not involved in metabolism
B. Second-Generation Antipsychotics		
Aripiprazole	Oxidation N-dealkylation	CYP2D6 and CYP3A
Amisulpiride	Oxidation (about 4%).	Underexplored
Asenapine	Glucuronization Oxidation Demethylation	CYP2D6 (the main path), CYP1A2, and CYP3A4
Ziprasidone	Oxidation	CYP3A4
Quetiapine	Oxidation	CYP3A4 and CYP2D6
Clozapine	Oxidation	CYP1A2
Lurasidone	Oxidation N-dealkylation	CYP3A4
Olanzapine	Oxidation	CYP2D6 and CYP1A2
Paliperidone	No hepatic metabolism. Excreted through the kidneys	Not involved in metabolism
Risperidone	Oxidation	CYP2D6 and CYP3A
Sertindol	Oxidation	CYP3A4 and CYP2D6

**Table A6:** Transporter proteins and their encoding genes.

Gene (OMIM * Number)	Chromosome Location **	Genomic Coordinate **	Protein ***
*ABCB1* (171050)	7q21.12	chr7:87,503,017–87,713,323(GRCh38/hg38)	Multidrug Resistance Protein 1 (MDR1) P-Glycoprotein 1 (P-gp)
*ABCB2* (170260)	6p21.32	chr6:32,845,209–32,853,816(GRCh38/hg38)	Transporter 1 (TAP1)
*ABCB3* (170261)	6p21.32	chr6:32,821,831–32,838,770(GRCh38/hg38)	Transporter 2 (TAP2)
*ABCB4* (171060)	7q21.12	chr7:87,398,988–87,480,435(GRCh38/hg38)	Multidrug Resistance Protein 3 (MDR2) P-Glycoprotein 3
*ABCB11* (603201)	2q31.1	chr2:168,915,468–169,031,396(GRCh38/hg38)	Bile Salt Export Pump (BSEP)
*ABCC1* (158343)	16p13.11	chr16:15,949,138–16,143,257(GRCh38/hg38)	Protein associated with multidrug resistance (MRP1)
*ABCC2* (601107)	10q24.2	chr10:99,782,602–99,853,741(GRCh38/hg38)	Multidrug Resistance-Associated Protein 2 (MRP2)
*ABCC3* (604323)	17q21.33	chr17:50,634,777–50,692,253(GRCh38/hg38)	Multidrug Resistance-Associated Protein 3 (MRP3)
*ABCC4* (605250)	13q32.1	chr13:95,019,835–95,301,475(GRCh38/hg38)	Multidrug Resistance-Associated Protein 4 (MRP4)
*ABCC5* (605251)	3q27.1	chr3:183,919,934–184,018,010(GRCh38/hg38)	Multidrug Resistance-Associated Protein 5 (MRP5)
*ABCC6* (603234)	16p13.11	chr16:16,149,565–16,223,617(GRCh38/hg38)	Multidrug Resistance-Associated Protein 6 (MRP6)
*ABCC1* (612509)	6p21.1	chr6:43,427,366–43,451,994(GRCh38/hg38)	Multidrug Resistance-Associated Protein 7 (MRP7)
*ABCC11* (607040)	16q12.1	chr16:48,164,842–48,249,973(GRCh38/hg38)	Multidrug Resistance-Associated Protein 8 (MRP8)
*ABCC12* (607041)	16q12.1	chr16:48,080,882–48,156,018(GRCh38/hg38)	Multidrug Resistance-Associated Protein 9 (MRP9)
*ABCG2* (603756)	4q22.1	chr4:88,090,150–88,231,628(GRCh38/hg38)	Breast cancer resistance protein (BCRP)

Note: * from the open database OMIM—Online Mendelian Inheritance in Man [78], ** from the open database GeneCards: The Human Gene Database [86], *** from the open database The Human Protein Atlas [81], *ABCB1*—ATP Binding Cassette Subfamily B Member 1, *ABCG2*—ATP-Binding Cassette Sub-family G member 2, and *ABCC1*—ATP Binding Cassette Subfamily C Member 1.

**Table A7:** Antipsychotics—substrates of transport proteins.

P-Glycoprotein (P-gp)	Breast Cancer Resistance Protein (BCRP)	Multidrug Resistance-Associated Protein 1 (MRP1)
Amisulpride Aripiprazole Asenapine Chlorpromazine Chlorprothixene Clozapine Fluphenazine Flupentixol Olanzapine Paliperidone Periciazine Quetiapine Risperidone Sertindole Sulpiride Trifluoperazine Ziprasidone Zuclopenthixol	Aripiprazole Chlorpromazine Clozapine Haloperidol Olanzapine Paliperidone Quetiapine Risperidone Sulpiride	Clozapine

**Table A8:** Candidate genes encoding targets/receptors of antipsychotics.

Gene (OMIM * Number)	Chromosome Location **	Genomic Coordinate **	Protein ***
Dopaminergic system			
*DRD2* (126450)	11q23.2	chr11:113,409,605–113,475,691(GRCh38/hg38)	Dopaminergic receptor type D2
*DRD3* (126451)	3q13.31	chr3:114,127,580–114,199,407(GRCh38/hg38)	Dopaminergic receptor type D3
*DRD4* (126452)	11p15.5	chr11:637,269–640,706(GRCh38/hg38)	Dopaminergic receptor type D4
Serotoninergic system			
*HTR2A* (182135)	13q14.2	chr13:46,831,546–46,898,082(GRCh38/hg38)	Serotonergic 5-hydroxytryptamine receptor type 2A
*HTR2C* (312861)	Xq23	chrX:114,584,078–114,910,061(GRCh38/hg38)	Serotonergic 5-hydroxytryptamine receptor type 2C
Glutamatergic system			
*GRIN2A* (138253)	16p13.2	chr16:9,753,404–10,182,928(GRCh38/hg38)	Glutamate ionotropic receptor type 2A
*GRIN2B* (138252)	12p13.1	chr12:13,437,942–13,982,134(GRCh38/hg38)	Glutamate ionotropic receptor type 2B

**Note:** * from the open database OMIM—Online Mendelian Inheritance in Man [78], ** from the open database GeneCards: The Human Gene Database [86], *** from the open database The Human Protein Atlas [81], *DRD2*—Dopamine Receptor D2, *DRD3*—Dopamine Receptor D3, *DRD4*—Dopamine Receptor D4, *HTR2A*—5-Hydroxytryptamine Receptor 2A, *HTR2C*—5-Hydroxytryptamine Receptor 2C, *GRIN2A*—Glutamate Ionotropic Receptor NMDA Type Subunit 2A, and *GRIN2B*—Glutamate Ionotropic Receptor NMDA Type Subunit 2B.

**Table A9:** Candidate genes encoding key targets/enzymes of antipsychotics in the central nervous system.

Gene (OMIM * Number)	Chromosome Location **	Genomic Coordinate **	Protein ***
*HSPG2* (142461)	1p36.12	chr1:21,822,244–21,937,310(GRCh38/hg38)	Perlecan
*COMT* (116790)	22q11.21	chr22:19,941,371–19,969,975(GRCh38/hg38)	Catechol-O-methyltransferase
*NQO1* (125860)	16q22.1	chr16:69,706,996–69,726,668(GRCh38/hg38)	Quinone dehydrogenase nicotinamide adenine dinucleotide phosphate
*RGS2* (600861)	1q31.2	chr1:192,809,039–192,812,275(GRCh38/hg38)	G protein signal transduction regulator 2
*GSTP1* (134660)	11q13.2	chr11:67,583,742–67,586,656(GRCh38/hg38)	Glutathione-S-transferase pi 1
*PPP1R1B* (604399)	17q12	chr17:39,626,707–39,636,626(GRCh38/hg38)	Regulatory inhibitor of subunit 1B of protein phosphatase 1
*BDNF* (113505)	11p14.1	chr11:27,654,893–27,722,058(GRCh38/hg38)	Brain-derived neurotrophic factor
*MnSOD* (147460)	6q25.3	chr6:159,669,069–159,762,281(GRCh38)	Manganese superoxide dismutase 2

Note: * from the open database OMIM—Online Mendelian Inheritance in Man [78], ** from the open database GeneCards: The Human Gene Database [86], *** from the open database The Human Protein Atlas [81], *HSPG2*—heparan Sulfate Proteoglycan 2, *COMT*—catechol-O-methyl transferase, *NQO1*—NAD(P)H quinone dehydrogenase 1, RGS2—regulator of G protein signaling 2, *GSTP1*—glutathione S-transferases P1, *PPP1R1B*—protein phosphatase 1 regulatory inhibitor subunit 1B, *BDNF*—brain-derived neurotrophic factor, and *MnSOD*—manganese superoxide dismutase.
